# NMDA receptor‐dependent and ‐independent effects of natural compounds and crude drugs on synaptic states as revealed by drebrin imaging analysis

**DOI:** 10.1111/ejn.15231

**Published:** 2021-04-27

**Authors:** Noriko Koganezawa, Yuko Sekino, Hitomi Kawakami, Hiroyuki Fuchino, Nobuo Kawahara, Tomoaki Shirao

**Affiliations:** ^1^ Department of Neurobiology and Behavior Gunma University Graduate School of Medicine Maebashi Japan; ^2^ Endowed Laboratory of Human Cell‐Based Drug Discovery Graduate School of Pharmaceutical Sciences The University of Tokyo Bunkyo‐ku Japan; ^3^ Research Center for Medicinal Plant Resources National Institutes of Biomedical Innovation, Health and Nutrition Tsukuba Japan; ^4^ AlzMed, Inc Bunkyo‐ku Japan; ^5^ Present address: The Kochi Prefectural Makino Botanical Garden Kochi Japan

**Keywords:** dendritic spine, high‐content imaging analysis, high‐throughput screening, primary neuronal culture, synapse, synaptic plasticity

## Abstract

Effective drugs that can cure cognitive impairments remain elusive. Because synaptic dysfunction has been correlated with cognitive impairments, drug development to target synaptic dysfunction is important. Recently, natural compounds and crude drugs have emerged as potential therapeutic agents for cognitive disorders. However, their effects on synaptic function remain unclear, because of lack of evaluation system with high reproducibility. We have recently developed highly reproducible in vitro high‐content imaging analysis system for evaluation of synaptic function using drebrin as a marker for synaptic states. Therefore, we aimed to examine the direct effects of well‐known natural compounds and crude drugs on synaptic states using this system. Rat hippocampal neurons were treated using natural compounds (nobiletin, diosgenin and tenuifolin) and crude drugs (Uncaria Hook [UH], Bezoar Bovis [BB], Coptis Rhizome [CR], Phellodendron Bark [PB] and Polygala Root [PR]). Immunocytochemical analysis was performed, and dendrite lengths and drebrin cluster densities were automatically quantified. We found that diosgenin, tenuifolin, CR, PB and PR decreased drebrin cluster densities, and the effects of PB and PR were partially dependent on N‐methyl‐D‐aspartic acid‐type glutamate receptors (NMDARs). Nobiletin and UH did not show any effects, whereas low‐dose BB treatment increased drebrin cluster densities. Our results showed that diosgenin, tenuifolin, BB, CR, PB and PR appeared to directly change synaptic states. Particularly, the NMDAR dependency of PB and PR appears to affect synaptic plasticity.

## Graphical abstract

Natural compounds and crude drugs have emerged as potential therapeutic agents for cognitive disorders. We used in vitro high‐content imaging analysis for cultured hippocampal neurons and analysed drebrin cluster density to evaluate synaptic function. Phellodendron Bark and Polygala Root decreased drebrin cluster density in an N‐methyl‐D‐aspartic acid‐type glutamate receptor (NMDAR)‐dependent manner, and the NMDAR dependency of these drugs appears to affect synaptic plasticity. Our assay is a promising method to examine natural compounds that affect synaptic states.

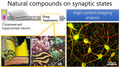

**Abbreviations**
AD: Alzheimer's disease
ANOVA: analysis of variance
BB: Bezoar Bovis
CR: Coptis Rhizome
DAPI: 4′,6‐diamidino‐2‐phenylindole, dihydrochloride
DIV: days in vitro
LTD: long‐term depression
LTP: long‐term potentiation
NMDAR: N‐methyl‐D‐aspartic acid‐type glutamate receptor
PB: Phellodendron Bark
PBS: phosphate‐buffered saline
PBSA: 3% bovine serum albumin in PBS
PR: Polygala Root
*SEM*
: standard error of the mean
UH: Uncaria Hook

## 1 INTRODUCTION

Currently, effective drugs that can cure cognitive impairments, such as those observed during Alzheimer's disease (AD), remain elusive. The pathology of AD includes the extracellular accumulation of amyloid‐beta peptide, the intracellular accumulation of hyper‐phosphorylated tau, and the loss of neuronal cells, particularly in the cerebral cortex and hippocampus. Among these symptoms, synaptic dysfunction has been correlated with cognitive dysfunction in AD patients. Therefore, the development of drugs that target synaptic dysfunction is important.

Recently, natural compounds and crude drugs have emerged as potential therapeutic agents for the treatment of cognitive dysfunction. Nobiletin, a citrus flavonoid, diosgenin, a well‐known steroidal sapogenin from wild yam, and tenuifolin, a bio‐active terpenoid from *Polygala tenuifolia*, have been well‐studied, especially in relation to AD (Liu et al., 2015; Lv et al., 2009; Matsuzaki et al., 2006; Nakajima & Ohizumi, 2019; Onozuka et al., 2008; Tohda et al., 2012; Wang, Jin, et al., 2019; Youn et al., 2019). Uncaria Hook (UH), Bezoar Bovis (BB), Coptis Rhizome (CR), Phellodendron Bark (PB) and Polygala Root (PR) are also well‐known for their effects on cognitive dysfunction and AD (Cai et al., 2013; Hu et al., 2014; Kalalian‐Moghaddam et al., 2013; Kanno et al., 2013; Ou et al., 2018; Park et al., 2002; Zhao et al., 2019, 2016). CR, PB and PR have previously been examined in clinical studies (Linn et al., 2012; Shin et al., 2009). UH, CR, PB and PR are plant‐based, and BB is an animal‐derived crude drug. However, many previous reports have focused on neuroprotective effects, such as anti‐neuroinflammatory or anti‐cell death effects, but did not examine direct effects on synaptic function, likely because few in vitro evaluation systems are capable of evaluating synaptic function, which underlies learning and memory.

We have recently developed an in vitro high‐content imaging analysis system for the evaluation of synaptic function, with a focus on drebrin (Hanamura et al., 2019). Drebrin is an actin‐binding protein that stabilizes actin filaments (reviewed in Koganezawa et al., 2017). Drebrin‐decorated stable actin filaments accumulate in dendritic spines and are thought to play an important role in synaptic plasticity, such as long‐term potentiation (LTP) and long‐term depression (LTD). The exodus of drebrin from dendritic spines, resulting in reduced drebrin cluster densities, occurs during the initial stages of synaptic plasticity, and is elicited by Ca^2+^ influx through the N‐methyl‐D‐aspartic acid‐type glutamate receptor (NMDAR) (Mizui et al., 2014; Sekino et al., 2006). Thus, drebrin can serve as a marker for synaptic states. Therefore, the detection of drebrin cluster reductions elicited by drug treatment indicates that the drug may stimulate the synapse and cause changes in synaptic states.

In this study, using the in vitro high‐content imaging analysis system, we investigated whether well‐known natural compounds and crude drugs can directly affect synaptic states. We examined three natural compounds (nobiletin, diosgenin and tenuifolin) and five crude drugs (UH, BB, CR, PB and PR).

## 2 MATERIALS AND METHODS

### 2.1 Animals

Animal experiments were performed according to the ARRIVE guidelines and the guidelines of the Animal Care and Experimentation Committee (Gunma University, Showa Campus, Maebashi, Japan) and conformed with the National Institutes of Health (NIH) guidelines for the use of animals in research. All efforts were made to minimize animal suffering and the number of animals used in this study. Timed pregnant Wistar rats (the 13th day of pregnancy) were obtained from Charles River Japan Inc. (Yokohama, Japan). The rats were maintained under standard animal facility conditions, with food and water available ad libitum.

### 2.2 Hippocampal neuronal cultures from frozen stocks

Pregnant Wistar rats were deeply anesthetized with isoflurane (Mylan N.V.) and killed by cervical dislocation. The hippocampi were dissected from the foetuses, at embryonic day 18, and the hippocampal cells were prepared according to previously described methods (Takahashi et al., 2003). Frozen stocks of hippocampal neurons, which is now commercially available as SKY Neuron (AlzMed, Inc.), were stored in liquid nitrogen, until use. Primary hippocampal cultures from frozen stock were prepared, in a manner similar to previously described methods (Hanamura et al., 2019; Mitsuoka et al., 2019). Briefly, the frozen stock was thawed in a thermostat bath, at 37°C for 3 min, and the neurons were plated on 96‐well plates (Gifted from Zeon Corp.; 3.0 × 10^4^ cells/cm^2^) that were pre‐coated with poly‐L‐lysine. The neurons were incubated in Neurobasal Medium, containing B‐27 supplement (50×), penicillin/streptomycin (100×) and L‐alanyl‐L‐glutamine (400×, Glutamax‐I; Thermo Fisher Scientific), at 37°C, in a 5% CO_2_ atmosphere. At 21 days in vitro (DIV), the neurons were treated with the various drugs.

To reduce variations among experiments, we used frozen stocks of rat hippocampal neurons that were verified to meet a predefined criterion for drebrin cluster density (more than 0.4/µm, at 21 DIV). In some cases, we observed varied neuron numbers among conditions. Therefore, we recruited data with rates of change below 10%.

### 2.3 Drug treatments

Stock solutions of tenuifolin (CFN98157; Wuhan ChemFaces Biochemical Co., Ltd.), nobiletin and diosgenin, (N0871 and D1474, respectively; Tokyo Chemical Industry Co., Ltd.) were diluted in dimethyl sulfoxide (DMSO, 100 mM) to final concentrations of 1, 10, 100 and 1,000 µM, for each drug. The commercially acquired crude drugs [CR: NIB‐0013 (Sichuan, China), PB: NIB‐0249 (Japan), UH: NIB‐0405 (Guangxi, China), PR: NIB‐0260 (Shanxi, China)], were provided by the Japan Kampo Medicines Manufacturers Association (JKMA) and were deposited at the Tsukuba Division, Research Center for Medicinal Plant Resources (RCMPR), National Institutes of Biomedical Innovation, Health and Nutrition (NIBIOHN). Stock solutions of CR, PB, UH, PR and BB (Lot O‐229; Gifted from Mitsuboshi Pharmaceutical Co., Ltd.) were also diluted in DMSO (40 mg/ml), and further dilutions were performed prior to drug administration. The final concentrations of these crude drugs were 0.4, 4, 40 and 400 µg/ml, and for some experiments, 80, 200, 300 and 800 µg/ml PR was used. The final concentration of DMSO for all experiments was either 1% or 0.1%. The drugs were administered 10 min before fixation. Some neurons were treated with 2‐amino‐5‐phosphonovaleric acid (APV, an NMDAR competitive antagonist, 100 µM, R&D SYSTEMS, TOCRIS) for 10 min, before the administration of drug treatments.

### 2.4 Immunocytochemistry

Cultured hippocampal neurons were fixed with 4% paraformaldehyde, in 0.1 M phosphate buffer. After permeabilization with 0.1% Triton X‐100 in phosphate‐buffered saline (PBS) for 5 min, the neurons were blocked with 3% bovine serum albumin in PBS (PBSA), for 1 hr at room temperature (RT). The neurons were then incubated overnight, at 4°C with primary antibodies. After washing with PBS, the neurons were incubated with the appropriate secondary antibodies and 4′,6‐diamidino‐2‐phenylindole, dihydrochloride (DAPI, 1:1,000, Thermo Fisher Scientific) in PBSA, for 1 hr at RT. The primary antibodies used in this study were anti‐drebrin antibody [mouse monoclonal, clone M2F6, hybridoma supernatant, (Shirao & Obata, 1986)] and anti‐MAP2 antibody (rabbit polyclonal, AB5622‐I, 1:2,000, Merck Millipore). The secondary antibodies used were Alexa Fluor 488 donkey anti‐mouse IgG (1:250, Jackson ImmunoResearch Laboratories, Inc.) and Alexa Fluor 594 donkey anti‐rabbit IgG (1:250, Jackson ImmunoResearch Laboratories, Inc.).

### 2.5 Automated image acquisition and analysis

**FIGURE 1:**
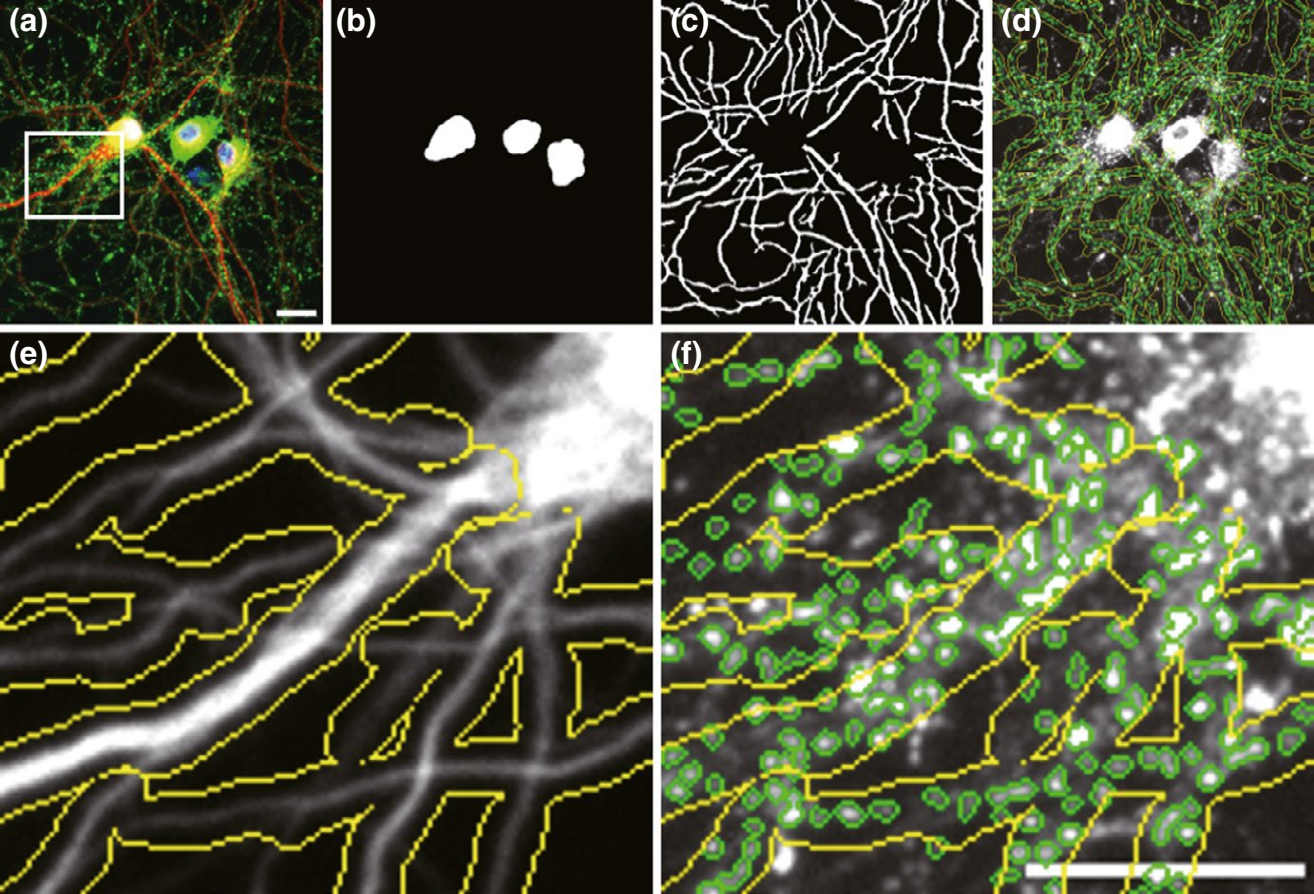
Quantification of neuronal cell bodies, dendrite and drebrin clusters by the IN Cell Developer Toolbox software. (a) Fluorescence images of MAP2 (red), drebrin (green) and DAPI (blue) in control neurons. (b) Neuronal cell bodies were recognized by nuclear (DAPI) and MAP2 staining. (c) Dendrites were recognized by the MAP2‐positive regions other than the neuronal cell bodies. (d) Drebrin clusters (green circles) were identified by drebrin staining around dendrites. (e,f) Higher magnification of a white rectangle in a. (e) MAP2 staining image with the area where the drebrin clusters are counted (yellow lines). Dilation commands (Kernel size = 7) were used to determine the defined distance to dendrites. (f) Drebrin staining image with the drebrin clusters recognized by the software. Drebrin‐positive puncta (green circles) located within the yellow line areas are counted as drebrin clusters along dendrites. Scale bars, 20 µm. DAPI, 4′,6‐diamidino‐2‐phenylindole, dihydrochloride

All fluorescence images of hippocampal neurons cultured in 96‐well plates were automatically acquired (20× lens, numerical aperture 0.45), using the automatic focus function of an IN Cell Analyzer 2200 (GE Healthcare). For image acquisition, 16 fields covering 7.1 × 10^6^ µm^2^, were selected from each well. All data were collected at 2,048 × 2,048 resolution, at 16 bits/pixel, and a single pixel in the image corresponded to a 325‐nm square in the specimen plane. The images were analysed by the IN Cell Developer Toolbox (GE Healthcare), and dendrite length, neuron number and the linear density of drebrin clusters along the dendrites were quantified. To identify the dendrites of neurons, dilation and erosion commands and segmentation function were used with distinct parameters for MAP2‐positive signals. The area with neuronal somata was subtracted. MAP2‐positive somata with a mean intensity of MAP2 signals >750 were counted as the neuron number. To identify drebrin clusters, the segmentation function localizes fluorescent objects based on fluorescence intensity of drebrin signals via a contrast‐based segmentation algorithm with distinct kernel size and sensitivity. In addition, only drebrin‐positive puncta located within a defined distance to the dendrite, which is determined by dilation commands, are counted as drebrin clusters along dendrites. Furthermore, drebrin‐positive puncta with a mean intensity of drebrin signals >1,500 were used for counting drebrin clusters along dendrites (Figure 1). The full details of this method were published previously (Hanamura et al., 2019).

### 2.6 Statistical analysis

All data were obtained from two independent experiments. GraphPad Prism8 (GraphPad Software) was used to perform statistical analysis, to draw the dose‐fitting curve and to calculate the half‐maximal effective concentration (EC_50_). The dose–response curves were fitted based on the following formula: *Y* = 1 + (Top − 1)/(1 + 10^[Log (EC50) −^ *^X^* ^]^; Top >0). For multiple comparisons, a one‐way analysis of variance (ANOVA), followed by Dunnett's test, was used, and the effects of APV were tested with a two‐way ANOVA. Data are reported as the mean ± standard error of the mean. Significance was set at *p* < 0.01.

## 3 RESULTS

### 3.1 Diosgenin and tenuifolin reduced drebrin cluster densities

Nobiletin, diosgenin and tenuifolin are natural compounds that have demonstrated protective effects against AD (Liu et al., 2015; Lv et al., 2009; Matsuzaki et al., 2006; Nakajima & Ohizumi, 2019; Onozuka et al., 2008; Tohda et al., 2012; Wang, Jin, et al., 2019; Youn et al., 2019). In the first series of experiments, we examined the effects of these natural compounds on synaptic states, using the high‐content imaging analysis. We treated neurons at 21 DIV with each drug, for 10 min.

**FIGURE 2:**
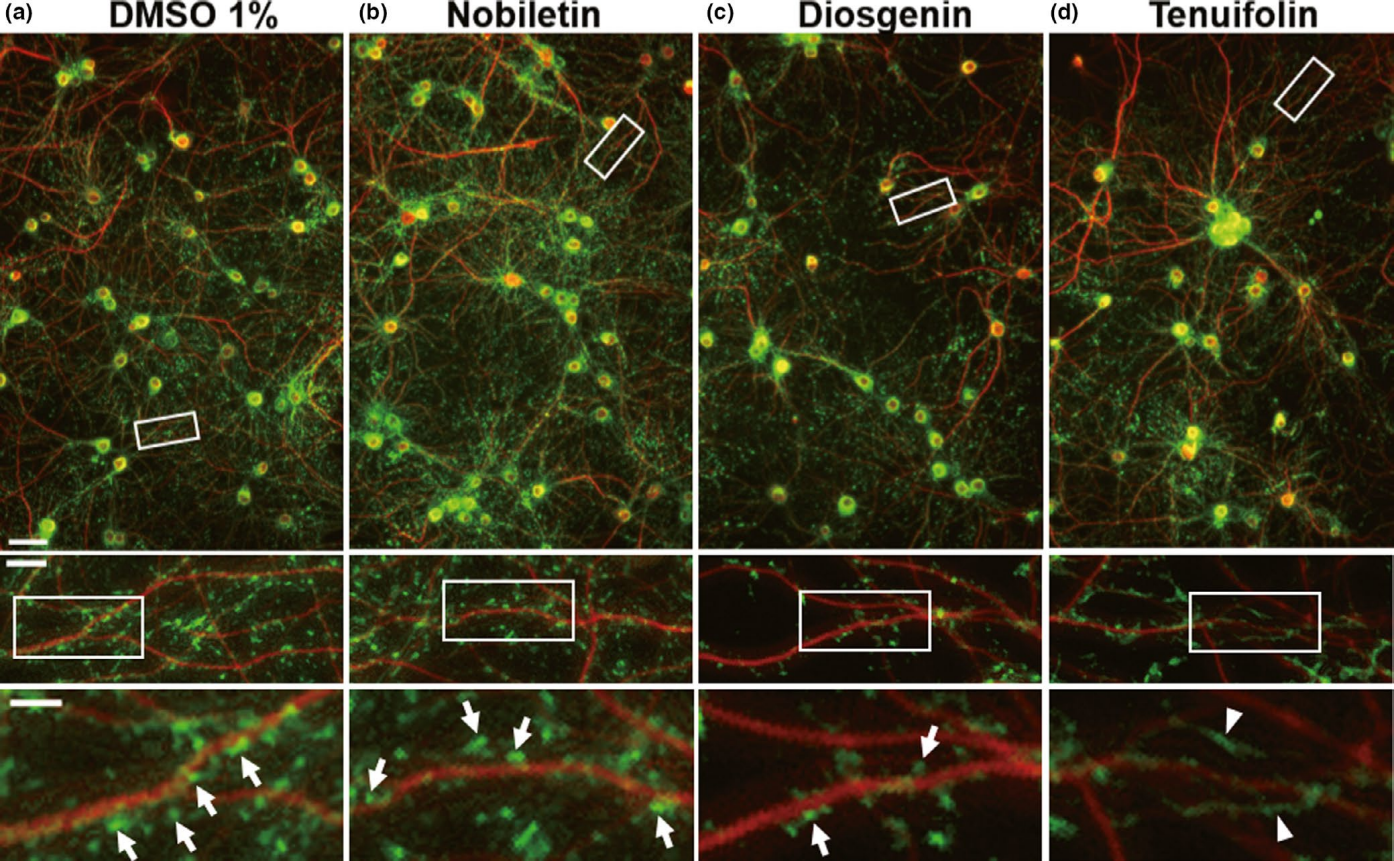
Representative images of neurons treated with natural compounds. Neurons were treated with 1% dimethyl sulfoxide (a), and 1,000 µM nobiletin (b), diosgenin (c) and tenuifolin (d). White rectangles indicate the positions of the images shown below. White arrows show drebrin clusters, and white arrowheads show the filamentous staining pattern of drebrin. Scale bars 50 µm (top image), 10 µm (middle image) and 5 µm (bottom image)

Figure 2a shows a representative image of neurons treated with 1% DMSO (sham treatment). Anti‐MAP2 and anti‐drebrin immunosignals are shown in red and green, respectively. As shown in the top panel of Figure 2a, the red and green signals colocalized within the cell somas, and MAP2‐positive dendrites can be observed. Drebrin puncta were observed along MAP2‐positive dendritic shafts, as shown in the middle panel of Figure 2a. The bottom panel of Figure 2a shows typical drebrin clusters (white arrows). Figure 2b shows neurons treated with 1,000 µM nobiletin, and the features of these images are similar to those observed for the sham treatment (Figure 2a). As shown in the bottom panel of Figure 2c, 1,000 µM diosgenin treatment decreased the drebrin‐immunopositive puncta. In addition, many filamentous drebrin immunostaining patterns appeared when 1,000 µM tenuifolin was applied (Figure 2d, bottom panel; white arrowheads), which is similar to the response observed following NMDAR stimulation using glutamate (Hanamura et al., 2019).

**FIGURE 3:**
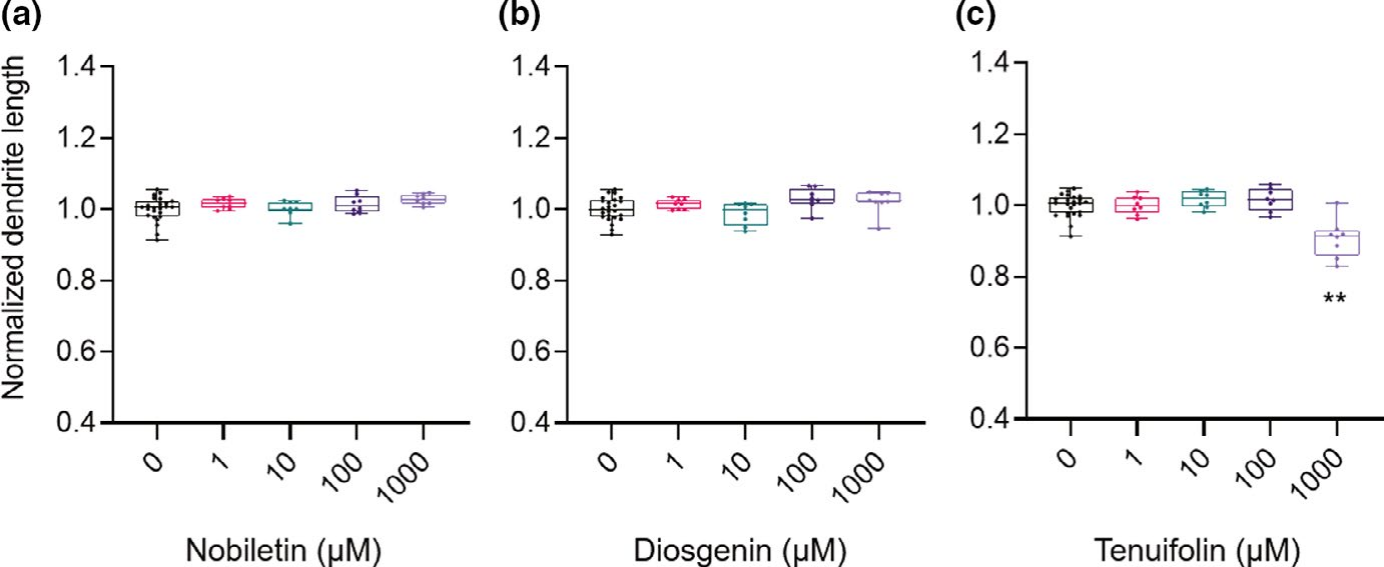
Quantitative data for normalized dendrite lengths. Nobiletin (a) and diosgenin (b) treatments did not display any effects on dendrite lengths, at any concentration. (c) treatment with 1,000 µM tenuifolin shortened dendrite lengths (one‐way ANOVA *p* < 0.0001, Dunnett's test *p* < 0.0001, 0 µM vs. 1,000 µM). 0 µM, *N* = 24 wells; 1, 10, 100, and 1,000 µM, *N* = 8 wells. **p < 0.01

**FIGURE 4:**
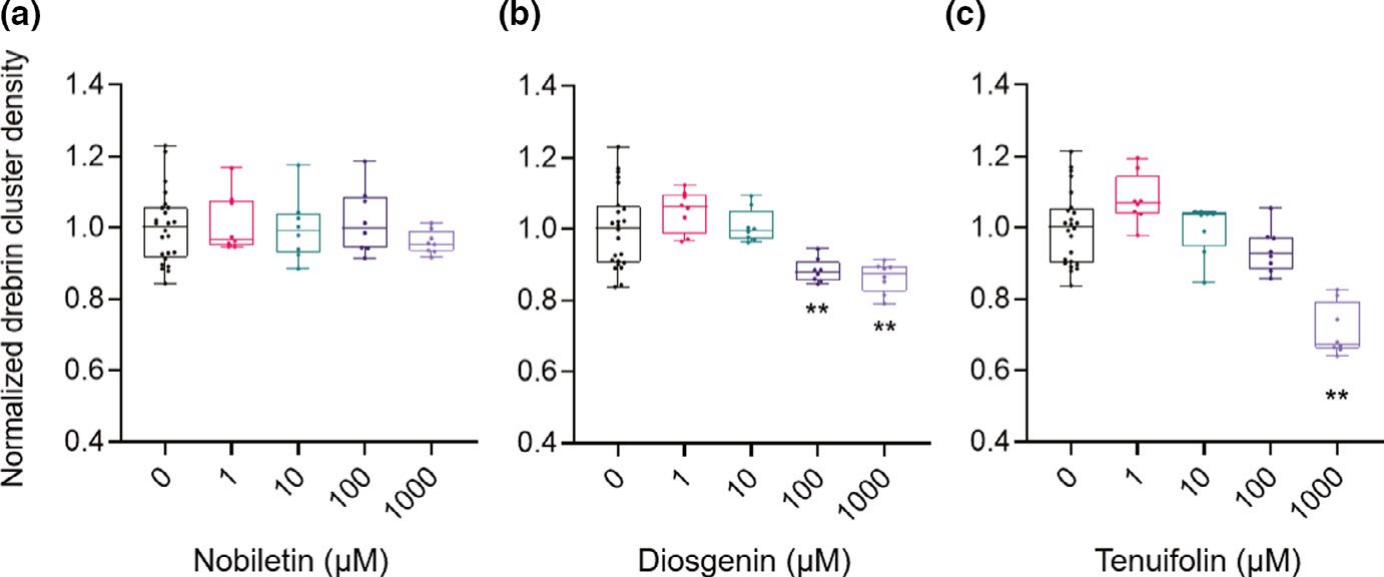
Quantitative data for normalized drebrin cluster densities. (a) Nobiletin treatments did not display any effects on drebrin cluster densities. (b) Diosgenin treatments decreased drebrin cluster densities at 100 and 1,000 µM (one‐way ANOVA *p* < 0.0001, Dunnett's test *p* = 0.003, 0 µM vs. 100 µM; *p* = 0.0006, 0 µM vs. 1,000 µM). (c) Treatment with 1,000 µM tenuifolin decreased drebrin cluster densities (one‐way ANOVA *p* < 0.0001, Dunnett's test *p* < 0.0001, 0 µM vs. 1,000 µM). 0 µM, *N* = 24 wells; 1, 10, 100, and 1,000 µM, *N* = 8 wells. **p < 0.01

Figure 3 shows the quantitative data for dendrite lengths (0 µM, *N* = 24 wells; 1, 10, 100, and 1,000 µM, *N* = 8 wells). Nobiletin and diosgenin did not affect the dendrite length, as shown in Figure 3a,b, respectively; however, 1,000 µM tenuifolin treatment shortened dendrite length by 9.2% ± 1.9% (*p* < 0.01; Figure 3c). Figure 4 shows the quantitative data for drebrin cluster densities (0 µM, *N* = 24 wells; 1, 10, 100, and 1,000 µM, *N* = 8 wells). Nobiletin treatment had no effects on drebrin cluster density (Figure 4a). Diosgenin and tenuifolin treatments both decreased drebrin cluster densities, in dose‐dependent manners (Figure 4b,c), with reductions of 14% ± 1.5% and 29% ± 2.5% for the highest doses of diosgenin and tenuifolin, respectively. To evaluate the differences in the effective concentrations for the reduction of drebrin cluster density among nobiletin, diosgenin, and tenuifolin, dose‐effective curves were generated for each drug. Using these curves, the half‐maximal effective concentration (EC_50_) values for each drug were determined. Nobiletin had almost no effects on drebrin cluster density reductions, whereas diosgenin and tenuifolin had EC_50_ values of 57.8 µM (95% confidential interval [CI] 14.7–220 µM) and 481 µM (95% CI 175–3,600 µM), respectively.

### 3.2 Reductions in drebrin cluster densities induced by diosgenin and tenuifolin did not depend on NMDAR

**FIGURE 5:**
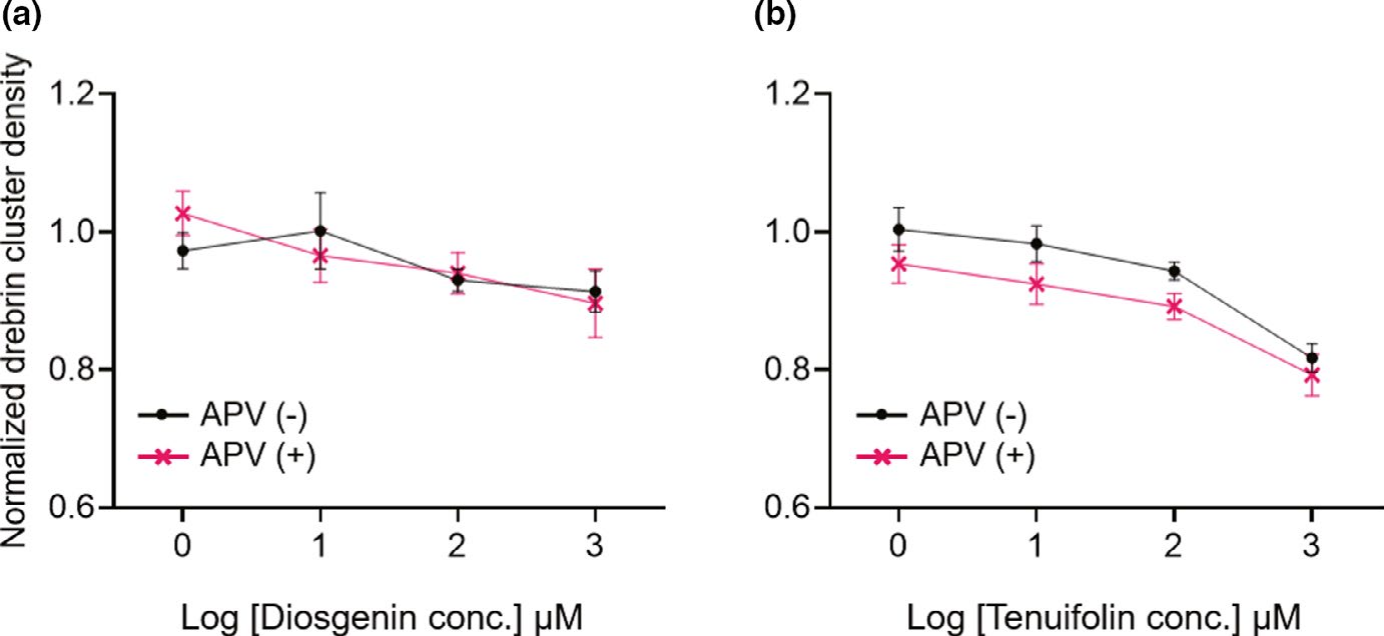
Quantitative data for normalized drebrin cluster densities after treatment with diosgenin (a; two‐way ANOVA, interaction, *p* = 0.76, dose comparison, *p* = 0.008, APV effects comparison, *p* = 0.80) or tenuifolin (b; two‐way ANOVA, interaction, *p* = 0.98, dose comparison, *p* < 0.0001, APV effects comparison, *p* = 0.08), either without APV (black line) or with APV (pink line) pre‐treatment. Both diosgenin and tenuifolin decreased drebrin cluster densities in dose‐dependent manners, but APV did not affect this response for either drug, indicating that NMDAR was not involved in these drebrin cluster density reductions. 0 µM, *N* = 18 wells; 1, 10, 100, and 1,000 µM, *N* = 8 wells. APV, 2‐amino‐5‐phosphonovaleric acid; NMDAR, N‐methyl‐D‐aspartic acid‐type glutamate receptor

Drebrin cluster density has been known to change in parallel with the intercellular Ca^2+^ concentration, and the activity‐dependent drebrin exodus caused by glutamate stimulation is NMDAR‐dependent (Mizui et al., 2014; Sekino et al., 2006). Therefore, we next investigated whether our observation of drebrin cluster reduction following diosgenin and tenuifolin treatments were NMDAR‐dependent. A 10‐min treatment with 100 µM APV was shown to block NMDAR‐dependent drebrin cluster reductions caused by 100 µM glutamate stimulation, to some extent, within this system (Mitsuoka et al., 2019). APV (100 µM) treatment was administered 10 min before the drug treatments. Interestingly, we did not observe any effects of APV treatments on the reduction of drebrin cluster densities (Figure 5a, diosgenin, two‐way ANOVA, APV effects comparison *p* = 0.80; Figure 5b, tenuifolin, two‐way ANOVA, APV effects comparison *p* = 0.08), suggesting that neither diosgenin nor tenuifolin decreased drebrin cluster densities in NMDAR‐dependent manners.

### 3.3 CR, PB and PR treatments decreased drebrin cluster densities

In the second series of experiments, we tested five crude drugs to determine whether these drugs could directly affect synaptic states. Stock solutions (40 mg/ml) of UH, BB, CR, PB and PR were diluted and applied to the neurons for 10 min.

**FIGURE 6:**
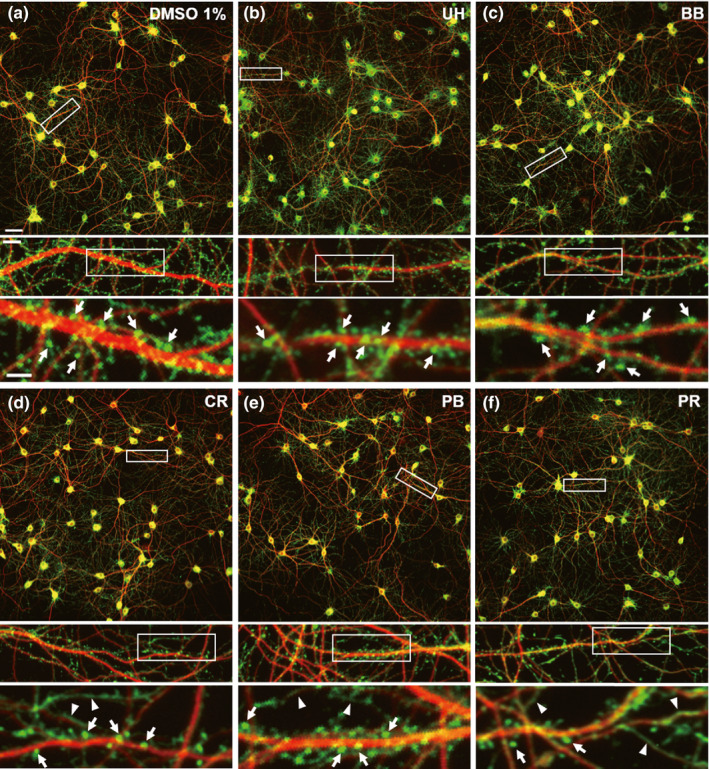
Representative images of neurons treated with crude drugs. (a) Neurons treated with 1% dimethyl sulfoxide. Neurons treated with 400 µg/ml UH (b), BB (c), CR (d), PB (e) and PR (f). White rectangles indicate the positions of images shown below. White arrows show drebrin clusters, and white arrowheads show the filamentous staining pattern of drebrin. Scale bars 50 µm (top image), 10 µm (middle image) and 5 µm (bottom image). BB, Bezoar Bovis; CR, Coptis Rhizome; PB, Phellodendron Bark; PR, Polygala Root; UH, Uncaria Hook

Figure 6a shows a representative image of neurons treated with 1% DMSO (sham treatment). As described for Figure 2a, drebrin puncta were observed along MAP2‐positive dendritic shafts (the middle panel of Figure 6a), and typical drebrin clusters can be observed in the bottom panel of Figure 6a (white arrows). Images from UH‐ and BB‐treated neurons showed similar features as sham‐treated neurons (Figure 6b,c, respectively). Careful observation indicated that treatments with the highest doses of CR and PB decreased drebrin puncta and increased the filamentous staining pattern of drebrin (the bottom panels of Figure 6d,e, respectively; white arrowheads). Similar to tenuifolin treatment, the highest dose of PR changed the drebrin staining pattern, and a filamentous staining pattern appeared (the bottom panel of Figure 6f; white arrowheads).

**FIGURE 7:**
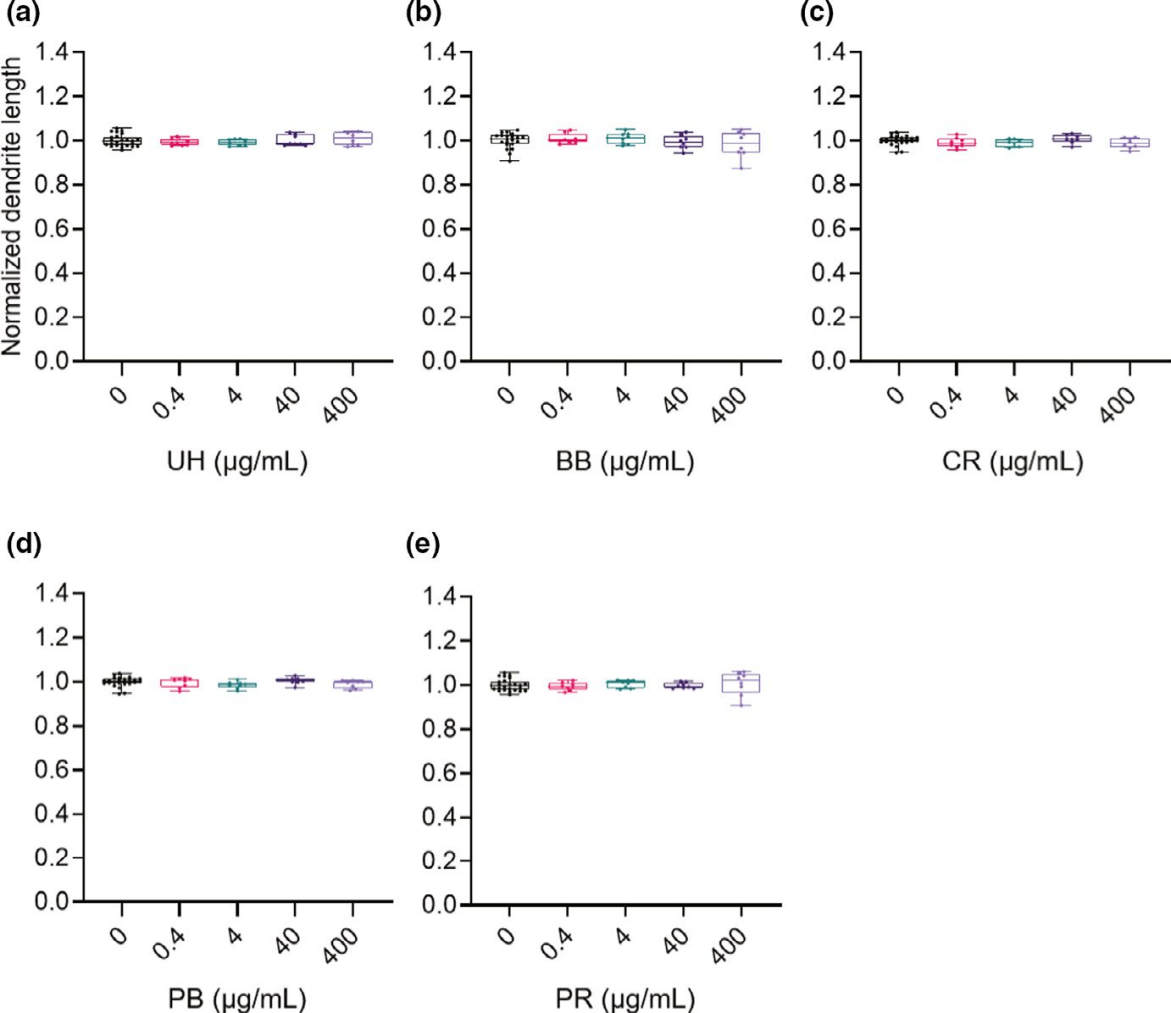
Quantitative data for normalized dendrite lengths (a, UH; b, BB; c, CR; d, PB; and e, PR). Dendrite lengths were not affected by treatment with any of the crude drugs. 0 µg/ml, *N* = 24 wells; 0.4, 4, 40, and 400 µg/ml, *N* = 8 wells. BB, Bezoar Bovis; CR, Coptis Rhizome; PB, Phellodendron Bark; PR, Polygala Root; UH, Uncaria Hook

**FIGURE 8:**
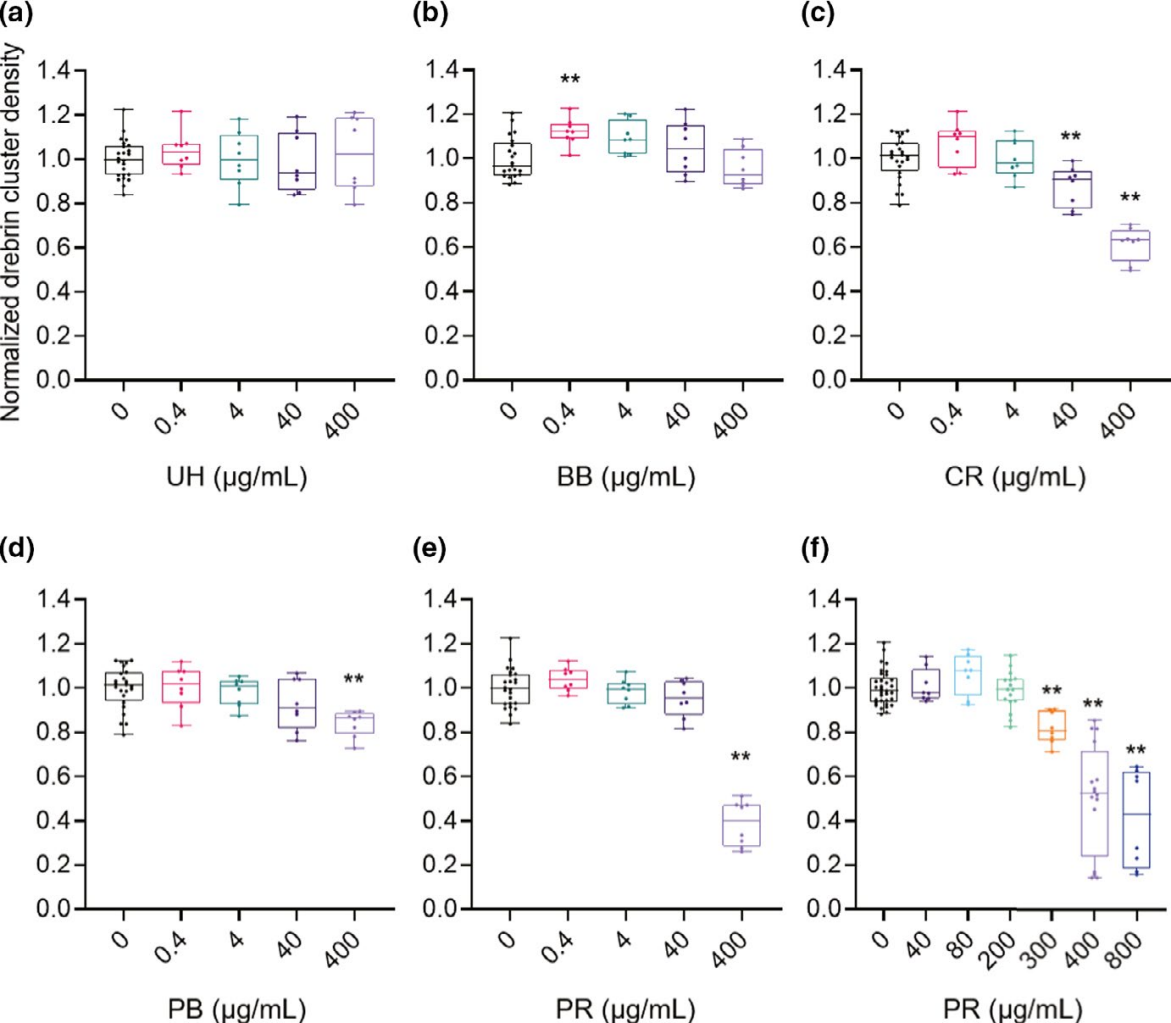
Quantitative data for normalized drebrin cluster densities. (a) UH treatments did not display any effects on drebrin cluster densities. (b) Low‐dose BB treatment (0.4 µg/ml) slightly but significantly increased drebrin cluster densities (one‐way ANOVA *p* = 0.001, Dunnett's test *p* = 0.008, 0 µg/ml vs. 0.4 µg/ml). (c) High‐dose CR treatments decreased drebrin cluster densities (one‐way ANOVA *p* < 0.0001, Dunnett's test *p* = 0.005, 0 µg/ml vs. 40 µg/ml; *p* < 0.0001, 0 µg/ml vs. 400 µg/ml). (d) High‐dose PB treatments decreased drebrin cluster densities (one‐way ANOVA *p* = 0.0008, Dunnett's test *p* = 0.0003, 0 µg/ml vs. 400 µg/ml). (e) High‐dose PR treatments decreased drebrin cluster densities (one‐way ANOVA *p* < 0.0001, Dunnett's test *p* < 0.0001, 0 µg/ml vs. 400 µg/ml). (f) Further PR treatments (40, 80, 200, 300, 400 and 800 µg/ml) were performed, and drebrin cluster densities were reduced in a dose‐dependent manner (one‐way ANOVA, *p* < 0.0001, Dunnett's test *p* = 0.001, 0 µg/ml vs. 300 µg/ml; *p* < 0.0001, 0 µg/ml vs. 400 µg/ml; *p* < 0.0001, 0 µg/ml vs. 800 µg/ml). (a–e) 0 µg/ml, *N* = 24 wells; 0.4, 4, 40 and 400 µg/ml, *N* = 8 wells. (f) 0 µg/ml, *N* = 33 wells; 200 and 400 µg/ml, *N* = 16 wells; 40, 80, 300 and 800 µg/ml, *N* = 8 wells. **p < 0.01. BB, Bezoar Bovis; CR, Coptis Rhizome; PB, Phellodendron Bark; PR, Polygala Root; UH, Uncaria Hook

Quantitative data (0 µg/ml, *N* = 24 wells; 0.4, 4, 40, and 400 µg/ml, *N* = 8 wells) showed that dendrite lengths did not change for any tested concentration of any drug (Figure 7). Drebrin cluster densities were also quantified (Figure 8; 0 µg/ml, *N* = 24 wells; 0.4, 4, 40, and 400 µg/ml, *N* = 8 wells). UH treatments did not have any effects on drebrin cluster densities (Figure 8a). The low‐dose treatment (0.4 µg/ml) of BB resulted in a 12% ± 2.1% increase in drebrin cluster density (*p* < 0.01; Figure 8b). CR (Figure 8c), PB (Figure 8d), and PR (Figure 8e,f) reduced drebrin cluster densities in dose‐dependent manners, with 38% ± 2.6%, 16% ± 2.0% and 59% ± 7.7% reductions observed, respectively, for the highest doses. To evaluate the differences in the effective concentrations required to reduce the drebrin cluster density among CR, PB and PR, the EC_50_ values for each drug were determined. CR, PB and PR had EC_50_ values of 85.7 µg/ml (95% CI 38.7–229 µg/ml), 26.1 µg/ml (95% CI 2.75–111 µg/ml) and 713 µg/ml (95% CI 447–848 µg/ml), respectively.

### 3.4 The effects of PB and PR partially depend on NMDAR

**FIGURE 9:**
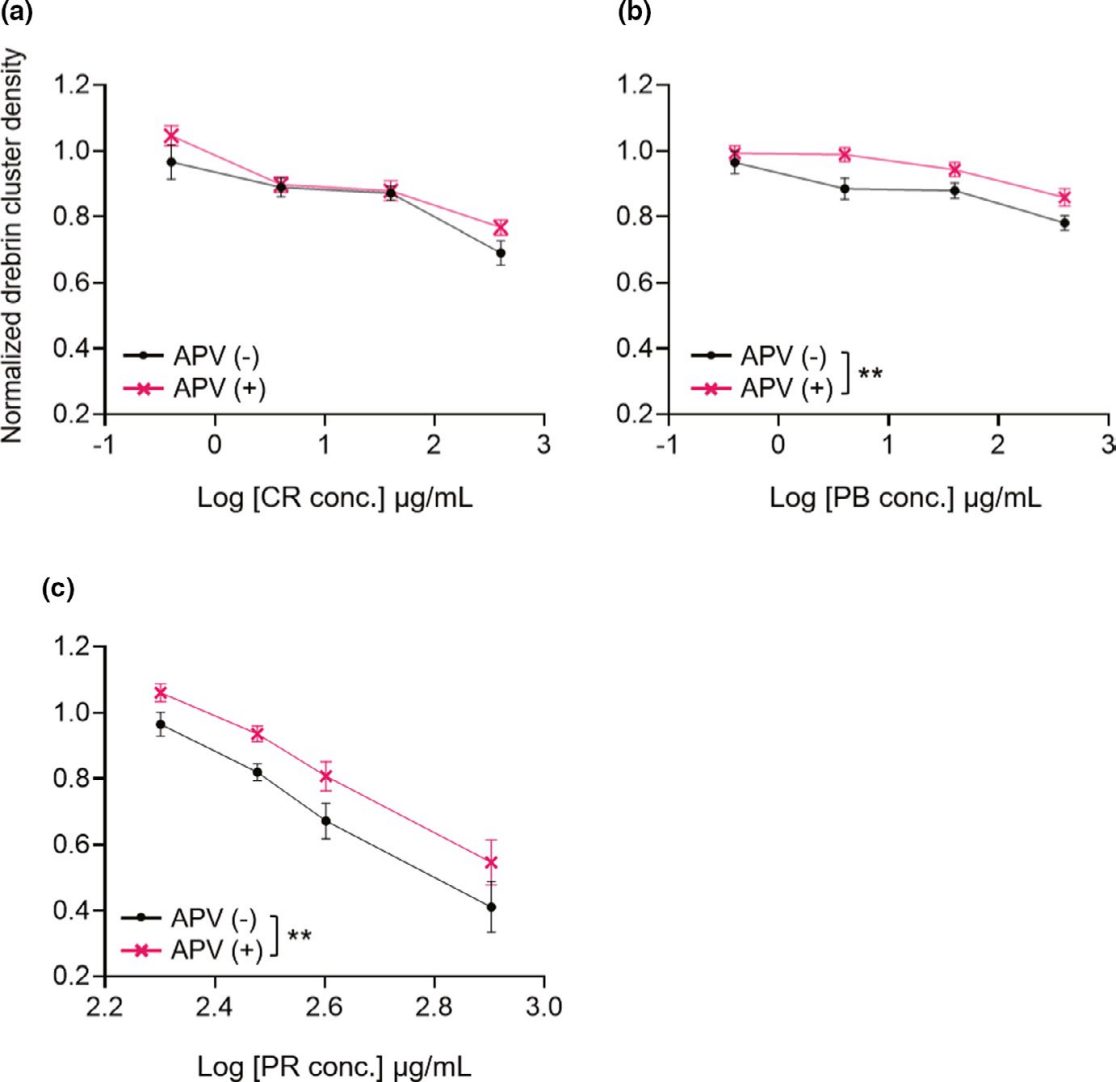
Quantitative data for normalized drebrin cluster densities following treatment with CR (a; two‐way ANOVA, interaction, *p* = 0.55, dose comparison, *p* < 0.0001, APV effects comparison, *p* = 0.061), PB (b; two‐way ANOVA, interaction, *p* = 0.57, dose comparison, *p* < 0.0001, APV effects comparison, *p* < 0.0001) or PR (c; two‐way ANOVA, interaction, *p* = 0.38, dose comparison, *p* < 0.0001, APV effects comparison, *p* = 0.0003), either without APV (black line) or with APV (pink line) pre‐treatment. CR, PB and PR decreased drebrin cluster densities in dose‐dependent manners; however, APV did not affect CR, whereas it did affect PB and PR. The effects of PB and PR on drebrin cluster density reduction partially depend on NMDAR. 0 µg/ml, *N* = 12 wells; 0.4, 4, 40, 200, 300, 400 and 800 µg/ml, *N* = 8 wells. **p < 0.01. APV, 2‐amino‐5‐phosphonovaleric acid; BB, Bezoar Bovis; CR, Coptis Rhizome; PB, Phellodendron Bark; PR, Polygala Root; UH, Uncaria Hook

We also investigated whether the cluster density reductions induced by CR, PB and PR treatment depended on NMDAR, using APV. In this experiment, we used the following concentrations; 0.4, 4, 40 and 400 µg/ml for CR and PB; and 200, 300, 400 and 800 µg/ml for PR. Drebrin cluster density reductions induced by CR treatments were not blocked by APV pre‐treatment (Figure 9a, two‐way ANOVA, APV effects comparison, *p* = 0.061), suggesting that CR reduces drebrin cluster densities in an NMDAR‐independent manner. However, the effects of APV on drebrin cluster density reductions were significant for the PB and PR treatments (Figure 9b, two‐way ANOVA, APV effects comparison, *p* < 0.0001; Figure 9c, two‐way ANOVA, APV effects comparison, *p* = 0.0003), suggesting that these drugs act in an NMDAR‐dependent manner.

## 4 DISCUSSION

Here, for the first time, we demonstrated that two natural compounds, diosgenin and tenuifolin, and four crude drugs, BB, CR, PB and PR, directly altered synaptic states. Among them, PB and PR decreased drebrin cluster densities in an NMDAR‐dependent manner. These two drugs may, therefore, affect synaptic plasticity because drebrin exodus following NMDAR activation occurs during the initial step of plasticity. Intriguingly, low‐dose BB treatment increased drebrin cluster density. Nobiletin and UH did not display any effects on drebrin cluster densities, suggesting that they do not change synaptic states. Furthermore, we determined the EC_50_ values for diosgenin, tenuifolin, CR, PB and PR effects on synaptic function, for the first time.

### 4.1 Both tenuifolin and PR decreased drebrin cluster densities, in different manners

While previous studies have shown that several PR extracts have neuroprotective activities (Cai et al., 2013; Hu et al., 2014; Park et al., 2002), we have found that both tenuifolin and PR have direct effects on synaptic states. Tenuifolin is a PR extract, which has been demonstrated to exert protective effects against amyloid‐beta toxicity (Liu et al., 2015; Wang, Jin, et al., 2019). Tenuifolin decreased drebrin cluster densities, without NMDAR involvement. Because tenuifolin is a saponin derived from PR, it may result in increased membrane permeability, elevating intracellular Ca^2+^ concentrations without requiring NMDAR activation (Zheng et al., 2019).

The drebrin cluster density reductions observed in response to PR treatments were stronger than those observed in response to tenuifolin treatments (59% ± 7.7% vs. 29% ± 2.5% reductions at the highest doses). This result suggests that other active compounds that affect NMDAR are included in PR, which is supported by the finding that the PR treatment effects were NMDAR‐dependent, whereas tenuifolin was NMDAR‐independent. Further analysis using the EC_50_ shift equation indicated that the APV effect on PR rejected the no‐shift hypothesis (*p* = 0.0005), suggesting that the dose–response curve of PR shifts to the right following APV treatments. Together, these results indicate that PR contains active compounds that impact synaptic plasticity. To clarify which components in PR affect NMDAR, the component analysis of PR is required.

### 4.2 Diosgenin decreased drebrin cluster densities, without involving NMDAR

Diosgenin has also been reported to have neuroprotective effects and to ameliorate cognitive deficits (Cheng et al., 2020; Chiu et al., 2011; Yang & Tohda, 2018), similar to PR and tenuifolin. We detected diosgenin effects on decreased drebrin cluster densities, even under NMDAR blockage conditions. Because diosgenin is a steroidal sapogenin, it may also increase membrane permeability, similar to tenuifolin, elevating intracellular Ca^2+^ concentrations and causing reductions in the drebrin cluster density. Tohda et al. reported that diosgenin can be a memory‐enhancing drug which mediates axonal growth in normal mice and AD model mice (Tohda et al., 2012, 2013). Therefore, we expected to observe changes in dendrite lengths; however, we did not observe any effects on dendrite length following the 10‐min treatment.

### 4.3 Both CR and PB include berberine but have different effects

CR and PB are well‐known to be stomachic, and their effects on the central nervous system have been vague (Wang, Wang, et al., 2019). However, in this study, these two drugs appear to have direct effects on synaptic states, suggesting their potential to act as therapeutic agents for AD.

The reduction in drebrin cluster densities was stronger following CR treatment than that following PB treatment (38% ± 2.6% vs. 16% ± 2.0% reductions at the highest dose). In addition, APV blocked PB slightly but did not block CR, suggesting that PB decreased drebrin cluster densities through NMDAR activation.

Berberine, a common extract found in these two drugs, has anti‐apoptotic properties, anti‐neuroinflammatory effects, and mitoprotective effects (Kalalian‐Moghaddam et al., 2013; Ou et al., 2018; Zhao et al., 2019). CR has been shown to contain more berberine than PB (Anetai, 1994; Anetai et al., 1987), which may result in a stronger effect on the reduction in drebrin cluster densities for CR compared with PB. Furthermore, berberine might be involved in the NMDAR‐independent mechanism of drebrin cluster reductions. Our data suggested that an active component other than berberine in PB has a direct effect on NMDAR activation.

### 4.4 Low dose of BB increased drebrin cluster density

To our surprise, the low‐dose treatment of BB increased drebrin cluster densities. BB has previously been demonstrated to improve the spatial learning and memory abilities in hyperlipemia vascular dementia model rats, via anti‐oxidative and cell‐protective effects (Zhong et al., 2016); however, whether BB treatment resulted in direct effects on synaptic function remained obscure.

BB has traditionally been used for the treatment of various diseases, including heart and liver disorders and high fever (reviewed in Yu et al., 2020). BB has also been used for the treatment of encephalopathy (Zhong et al., 2016). Bile acid, a major extract in BB, has been reported to suppress N‐type Ca^2+^ channel function (Lee et al., 2012), suggesting that BB affects synaptic states. Extracellular Ca^2+^ chelation has been shown to increase drebrin accumulation (Mizui et al., 2014). Thus, BB may increase drebrin cluster densities via the suppression of intracellular Ca^2+^ elevation. A detailed study remains necessary to clarify how BB works and to determine the effective concentration of BB on synaptic function.

### 4.5 Nobiletin and UH did not affect synaptic states

Nobiletin is a prospective compound for the treatment of AD and has been shown to rescue memory deficits in AD model animals (Matsuzaki et al., 2006; Onozuka et al., 2008). Nobiletin has also been reported to restore the MK‐801‐induced impairment of the NMDA‐stimulated phosphorylation of extracellular signal‐related kinase (Nakajima et al., 2007). In the present study, nobiletin did not affect synaptic states. Together, these results suggested that nobiletin does not affect NMDAR directly but, instead, has an antagonistic effect against MK‐801.

Some active components found in UH, such as isorhynchophylline, an oxyindole alkaloid, have been demonstrated to exert neuroprotective effects against glutamate‐induced neuronal death (Shimada et al., 1999). Although UH did not alter drebrin cluster densities in our study, UH might have inhibitory effects against NMDAR. Thus, the performance of an inhibitory assay against glutamate using our system may be useful (Mitsuoka et al., 2019).

### 4.6 PR and PB affect synaptic plasticity

In the present study, we showed that PR and PB decrease drebrin cluster densities through an NMDAR‐dependent manner, suggesting that these drugs elicited drebrin exodus. As previously mentioned, drebrin exodus occurs during the initial stage of synaptic plasticity, such as LTP and LTD. However, during LTP stimulation, the drebrin exodus is transient (Sekino et al., 2017). Whether these drugs induce LTP or LTD should be investigated by determining whether the drebrin exodus is transient following drug treatment, using the live‐cell imaging of GFP‐drebrin‐transfected neurons (Mizui et al., 2014).

In contrast, diosgenin, tenuifolin and CR decrease drebrin cluster densities through an NMDAR‐independent manner. To clarify the mechanism, further examination of their affinity to NMDAR and intracellular Ca^2+^ imaging might be useful.

### 4.7 Efficacy of the in vitro evaluation system for synaptic function

In our study, we used the recently developed in vitro high‐content imaging analysis system (Hanamura et al., 2019) to calculate the EC_50_ values for diosgenin, tenuifolin, CR, PB and PR for synaptic function, for the first time. This result suggests that our high‐content imaging analysis system can efficiently provide related data for various drug concentrations using one measurement. As demonstrated, this assay is relatively easy to perform, and has high‐throughput features and high reproducibility; therefore, it is suitable for the screening of chemicals including natural compounds and crude drugs. In this study, we were able to use this system to identify several candidates that may represent effective drugs for the treatment of cognitive dysfunction. This system represents a promising screening method for examining various chemicals that may affect synaptic function.

## 5 CONCLUSIONS

Our results showed that diosgenin, tenuifolin, BB, CR, PB and PR directly changed synaptic states, whereas the other tested compounds did not. Particularly, the NMDAR dependency of PB and PR appears to affect synaptic plasticity; therefore, these may represent promising drugs for synaptic dysfunction. Furthermore, the assay used is a promising screening method with high reproducibility for the identification of various chemicals that affect synaptic states.

## CONFLICT OF INTEREST

Tomoaki Shirao is CEO of AlzMed, Inc.

## AUTHOR CONTRIBUTIONS

N.Kg., Y.S., N.Kw. and T.S. designed the experiments; N.Kg. performed the experiments, analysed the data and wrote the manuscript; N.Kg., Y.S., H.K., H.F., N.Kw. and T.S. discussed the results and commented on the manuscript.

### PEER REVIEW

The peer review history for this article is available at https://publons.com/publon/10.1111/ejn.15231.

## Data Availability

Data related to this study are available from the corresponding author upon reasonable request.
